# Organic Vapour Sensing Properties of Area-Ordered and Size-Controlled Silicon Nanopillar

**DOI:** 10.3390/s16111880

**Published:** 2016-11-09

**Authors:** Wei Li, Zhilin Feng, Enwen Dai, Jie Xu, Gang Bai

**Affiliations:** 1 State-Province Joint Engineering Laboratory for RF Integration and Micropackaging, College of Electronic Science and Engineering, Nanjing University of Posts and Telecommunications, Nanjing 210023, China; 1215022733@njupt.edu.cn (Z.F.); 1015020833@njupt.edu.cn (E.D.); jiexu@njupt.edu.cn (J.X.); 2 State Key Laboratory of Electronic Thin Films and Integrated Devices, University of Electronic Science and Technology of China, Chengdu 610054, China; 3 Laboratory of Solid State Microstructures, Nanjing University, Nanjing 210093, China

**Keywords:** ordered silicon nanopillar, organic vapour, gas sensing

## Abstract

Here, a silicon nanopillar array (Si-NPA) was fabricated. It was studied as a room-temperature organic vapour sensor, and the ethanol and acetone gas sensing properties were detected with I-V curves. I-V curves show that these Si-NPA gas sensors are sensitive to ethanol and acetone organic vapours. The turn-on threshold voltage is about 0.5 V and the operating voltage is 3 V. With 1% ethanol gas vapour, the response time is 5 s, and the recovery time is 15 s. Furthermore, an evaluation of the gas sensor stability for Si-NPA was performed. The gas stability results are acceptable for practical detections. These excellent sensing characteristics can mainly be attributed to the change of the overall dielectric constant of Si-NPA caused by the physisorption of gas molecules on the pillars, and the filling of the gas vapour in the voids.

## 1. Introduction

In recent years, gas sensors have received much attention because of serious environmental pollution caused by the rapid development of modern industry [1,2,3,4,5,6]. As with most types of other sensors, gas sensors should also have high sensitivity and short response time, good long-term stability, and low operating voltage. Not only all kinds of gas sensitivity materials but also many micro-structures have been extensively studied, such as SnO_2_ [7,8], WO_3_ [9,10], grapheme [11,12], MoS_2_ [13,14], and carbon nanotube [15,16]. As found in detecting humidity, organic vapour gas sensors showed an improved sensitivity. Arena et al. developed flexible sensors for the detection of ethanol at room temperature by depositing sensitive layers consisting of ITO nanopowder dispersed into poly-diallyldimethylammonium chloride [17]. Wang et al. demonstrated that a ZnO nanorod gas sensor exhibited a high, reversible, and fast response to ethanol [18]. Tan et al. reported that a highly sensitive ethanol gas sensor was designed based on the mechanism of ethanol quasi-molecular imprinting [19]. Although these gas sensors demonstrated excellent performance, further development of Si-based material sensors—such as porous silicon [20,21] and silicon pillar [22,23]—is desirable, as it would make the assembly of the sensors much simpler, cheaper, and more portable because of their ease in integrating with the already-existing Si integrated circuit technology.

In our previously published papers, it has been reported that an effective pathway for gas transportation could be built in a silicon nanopillar array (Si-NPA), which was an ordered and morphology-controlled structure. Excellent humidity sensing properties, including high sensitivity and fast response rate, were achieved [22,23]. In this paper, the room-temperature organic vapour (ethanol and acetone gas) sensing properties of Si-NPA were studied, and the underlying mechanisms were analysed based on its unique surface structure, morphology, and physical properties. The results strongly indicated that Si-NPA might be a promising sensing material for future gas sensor applications.

## 2. Experiment

**Figure 1:**
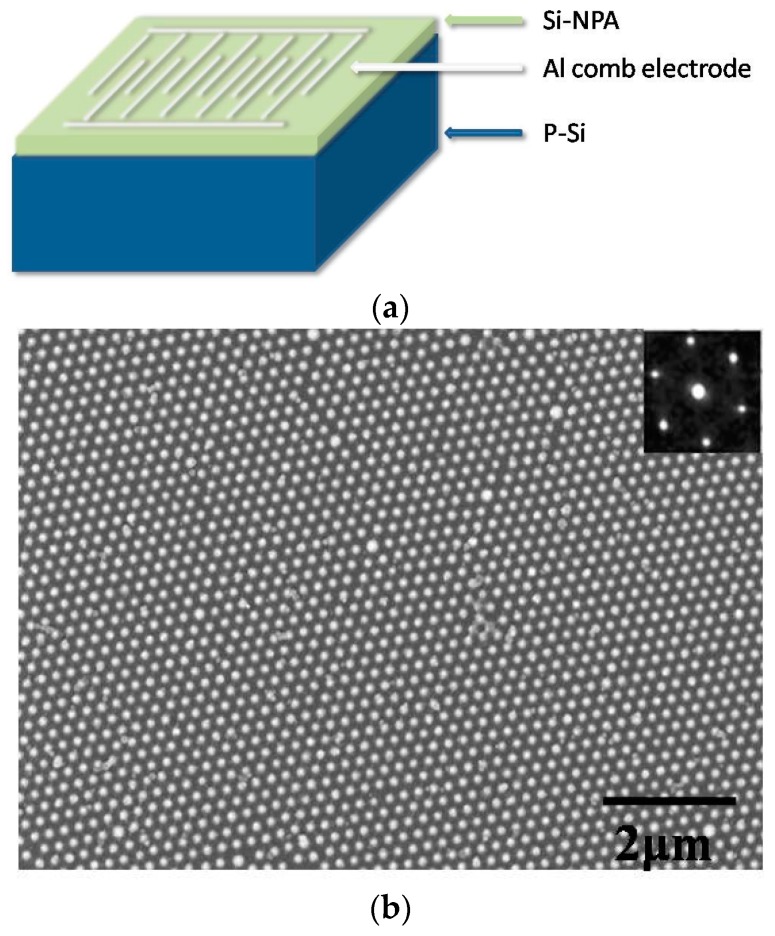

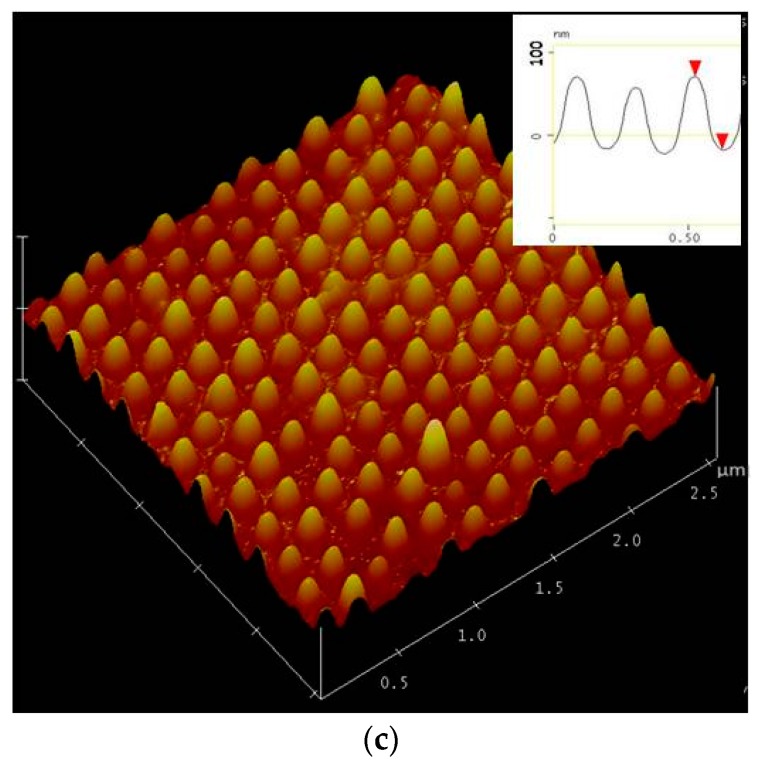
(**a**) Schematic diagram of the gas sensor; (**b**) Typical morphology scanning electron microscope (SEM) image of the silicon nanopillar array (Si-NPA); (**c**) Oblique view of an atomic force microscopy (AFM) image of the Si nanopillar array.

The ordered Si-NPA fabricated by nanosphere lithography [24,25] was studied as a new Si-based nanostructure material for gas sensors. By using this nanosphere lithography technique, an ordered and uniform Si nanopillar array can be obtained. The size of silicon nanopillars can be easily controlled by an oxidation and etching process. The period and density of nanopillar arrays are determined by the initial diameter of the polystyrene spheres. The gas sensor design is demonstrated in the schematic diagram presented in Figure 1a. The sensors were fabricated on 10 mm × 10 mm Si-NPA squares. The Si-NPA substrates were fabricated in four steps. First, a monolayer of PS nano-spheres with diameter of 220 nm were coated on the p-type (100) Si substrates by using the self-assembly technique; then, the coated substrates were etched in the reaction ion etching (RIE) system by using 20 sccm O_2_ gas under radio frequency (RF) power of 20 W. Next, the substrates were etched by using 40 sccm CF_4_ gas under RF power of 40 W; finally, the polystyrene (PS) nanospheres were removed in tetrahydrofuran (THF). The etching times in the second and third steps were 90 s and 4 min, respectively. An aluminum comb-like electrode was prepared by electron-beam evaporation (EBV) on the top side of the Si-NPA square. These electrodes were adopted to realize the formation of parallel plate and to ensure a large sensing area. The I-V curve measurement of the Si-NPA sensor was performed by placing the gas sensor in a chamber connected to a container of vapour mixtures by a tube. Organic gases were generated by evaporation from organic solution diluted from 0.25% to 1% concentrations by distilled water. Before exposure to organic vapours, the sensors were cleared with nitrogen gas, and then gas mixture containing these organic vapours was blown on the sensor surface. All these electrical measurements were carried out under atmospheric pressure and at room temperature.

## 3. Results and Discussions

The SEM and AFM measurement was carried out to investigate the structure and morphology of the silicon nanopillars. The SEM measurement was performed on LEO1530VP, and the AFM measurement was performed on a Nanoscope III (Digital Instrument, Tonawanda, NY, USA). The typical morphology of Si-NPA is presented in Figure 1b, which displays a classic honeycomb structure. As seen, well-separated, quasi-identical silicon pillars are clearly observed. In addition, the inset image gives the corresponding fast Fourier transform (FFT) pattern, which confirms that this array is regular hexagonal shape. In our experiment, the ordered area of Si-NPA is up to a few square centimetres. Figure 1c is an oblique view of an AFM image of the Si nanopillar array. As observed, all the pillars have distinct side walls and sharp edges. From the SEM and AFM images, the size of Si pillar is about 120.1 nm, and the height is 100 nm. The size of silicon nanopillars can be easily controlled by the etching process. The period and density of the nanopillar arrays are determined by the initial diameter of the polystyrene spheres. The silicon nanopillar array is 220 nm in period, and the density is ~10^9^/cm^2^.Obviously, the valleys around the pillar are intercommunicated and form an effective pathway for vapour transportation into or out of Si-NPA [22,23].

**Figure 2:**
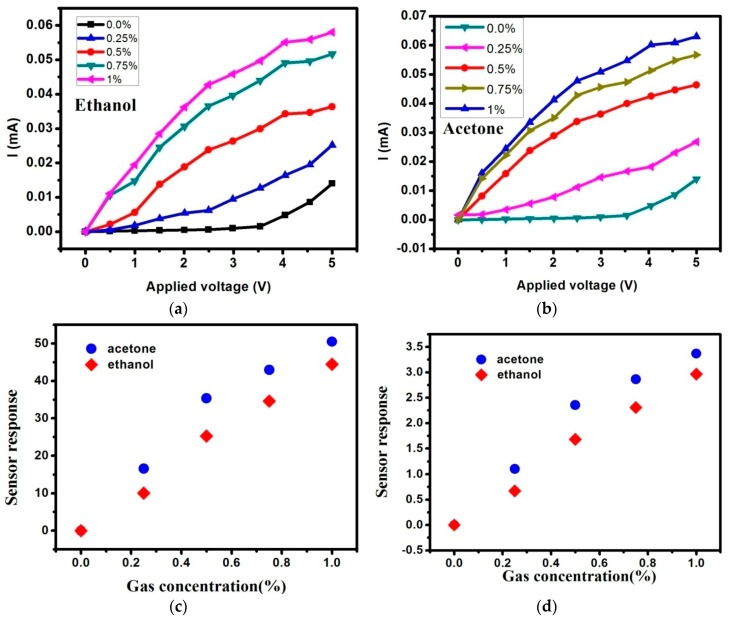
(**a**) I-V curves for ethanol concentration from 0%–1%; (**b**) I-V curves for acetone concentration from 0%–1%; (**c**) The sensor response versus gas concentration curves of the Si-NPA sensor with the applied voltage 3 V; (**d**) The response versus gas concentration curves of the Si-NPA sensor with the applied voltage 5 V.

The measurement of electrical properties was carried out with a semiconductor analyser to observe I-V curves at room temperature. In our previous work [22], it was found that the current had a more rapid increase at higher relative humidity (RH) with the same applied voltage. Consequently, this work is performed at 40% RH to avoid the effects of humidity. The current response was analysed for 0–5 V bias voltage with ethanol, and acetone vapours evaporated from 0% to 1% solution concentrations. Figure 2a shows the result of I-V curves for ethanol. As seen in this figure, the current increases rapidly. The turn-on threshold voltage is about 0.5 V (defined as the voltage to extract a current of 10 μA). This indicates that this gas sensor can work with low-power. At the applied voltage 3 V, the current raised from 1 μA to 45 μA when ethanol concentration increased from 0% to 1%, increasing over 45-fold. Additionally, it raised from 15 μA to 60 μA at the applied voltage 5 V, increasing by just over 4-fold. Furthermore, it seems that the current increased faster below 3 V. This result is due to the influence of the substrate with bias voltage. At a higher voltage (5 V), the current from the influence of the substrate is bigger. So, these results indicate that this gas sensor can work with low-power and it do well. The same measurements were carried out for acetone as performed for ethanol. The results also show similar sensing behaviour in I-V curves, as shown in Figure 2b. These results indicate that these Si-NPA gas sensors are sensitive to ethanol and acetone organic vapours.

The variation amplitudes of the current at various gas concentrations can be reflected more clearly by the function of sensor response (*K*):

(1) $$K=\frac{\Delta G}{G_{0}}$$

where ∆G =G_g_ − G_0_, G_g_ and G_0_ represent electric conductance in gas atmosphere and ambient air, respectively. The response versus gas concentration curves of the Si-NPA sensor with the applied 3 V and 5 V voltage are depicted in Figure 2c,d. Obviously, the gas sensors are more sensitive with an applied voltage of 3 V. The main reason for this is the influence of the substrate, as discussed above.

The current variation with gas concentration is generally suggested to be related to the change of the overall dielectric constant. Generally, the change of the overall dielectric constant mainly originates from the organic vapour molecular adsorption and gas vapour filling at low gas concentrations, and the adsorption of gas molecules can be classified as chemisorption and physisorption [26,27]. The occurrence of chemisorption requires relatively higher energy to exceed the adsorption activation energy [28]. In our experiments, all of the measurements were carried out at room temperature, and so the chemisorption of the gas molecules on Si-NPA seemed to be difficult. Therefore, the current variation of Si-NPA with gas concentration should be mainly attributed to the change of the overall dielectric constant of Si-NPA caused by the physisorption of gas molecules on the pillars and the filling of the gas vapour in the voids. The permittivity of ethanol and acetone are 24.3 and 20.7, which are larger than those of crystal silicon and ambient air. Additionally, more than 80% of the surface space of this gas sensor was filled with gas vapour. This strongly implies that the variation of the overall permittivity of Si-NPA is very large. In addition, the adsorption magnitude of gas vapour at the Si-NPA surface is different according to the surface state. It is well-known that the oxidation treatment of silicon surfaces has a considerable effect on the adsorption, due to the fact that HF-treated silicon layers are hydrophobic, while oxidized layers are hydrophilic. The gas sensors in this study are oxidized in nature, and therefore a high current response was observed for both gas vapours. So, this gas sensor is highly sensitive and it is even able to detect gas concentrations below 0.25%. Moreover, the gas sensors are more sensitive for acetone than ethanol, as shown in Figure 2c,d. This is due to the dipole moment of gas vapour. It has been reported that a many-fold current increment appears when exposing the sensing layers to organic vapours with large dipole moment [29]. In our case, the same results are found. The dipole moments of ethanol and acetone are 1.69 and 2.88, respectively. So, the dipole moment is another physical factor that affect the conductivity and sensor response.

**Figure 3:**
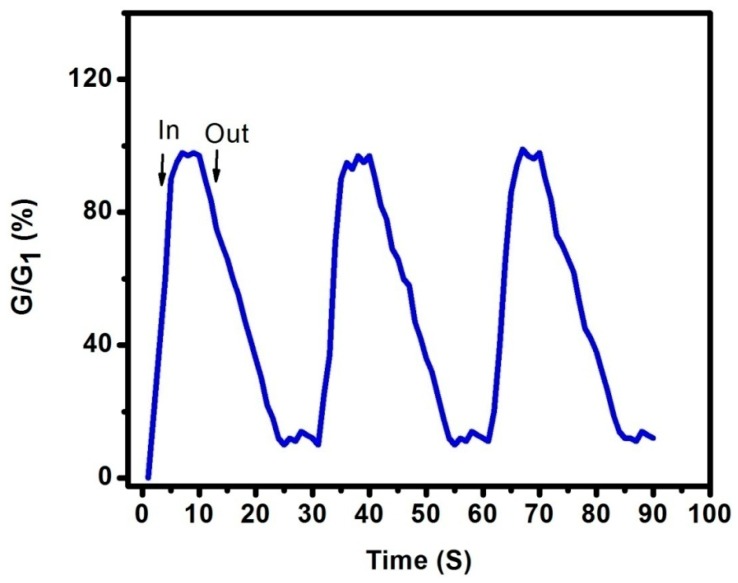
The response and recovery time at an applied voltage of 3 V with 1% ethanol gas vapour.

The response and recovery time were also studied. Figure 3 shows the response and recovery time at an applied voltage of 3 V with 1% ethanol gas vapour. G is the instantaneous conductance of this Si-NPA, and G_1_ is the final/initial values. The response time is defined as the time spent from G/G_1_ = 10% to 100% when the Si-NPA is taken into gas vapour. Instead, the recovery time is defined as the time spent from G/G_1_ = 100% to 10% when the Si-NPA is taken out from the gas vapour. As observed in this figure, the response time is 5 s, and the recovery time is 15 s. The faster response might due to the regular morphology. As has been seen in Figure 1c, the distinct-sidewall and well-separated silicon pillars were uniform. In addition, the quasi-identical and ordered honeycomb array was an effective channel, more suitable for the absorption and desorption of gas molecules. As a result, the response and recovery time are reduced.

**Figure 4:**
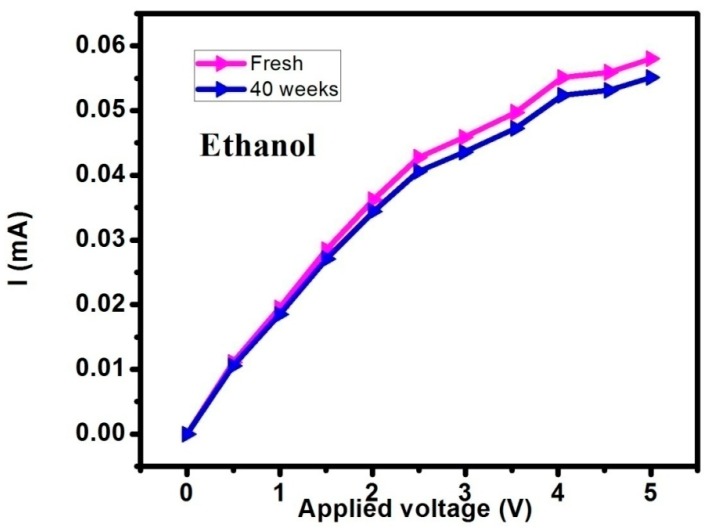
The ethanol measurement stability for Si-NPA.

The ethanol measurement stability for Si-NPA was performed after the sensor was exposed to air for 40 weeks, as shown in Figure 4. Compared with the freshly prepared samples, the curves measured after 40 weeks storage were changed. The current drift is about <5%.This is due to the active surface of Si-NPA, which could be oxidized. However, this gas stability result is acceptable for practical detection.

## 4. Conclusions

In conclusion, we have fabricated a room-temperature gas sensor based on a silicon nanopillar array, and the corresponding ethanol and gas acetone gas sensing properties were studied. I-V curves showing the gas sensitivity of the Si-NPA sensor were investigated with gas concentrations from 0% to 1%. The results showed that the turn-on threshold voltage for this Si-NPA sensor is just 0.5 V, and the operating voltage is 3 V. The response time was 5 s, and the recovery time was 15 s at an applied voltage of 3 V with 1% ethanol gas vapour. The ethanol measurement stability for Si-NPA was performed after the sensor was exposed to air for 40 weeks, and the current drift was about <5%. These results should be mainly attributed to the change of the overall dielectric constant of Si-NPA caused by the physisorption of gas molecules on the pillars, and the filling of the gas vapour in the voids. These excellent sensing characteristics indicate that Si-NPA might be expected to have important application in gas sensors.
